# ESF Workshop on ‘Sustainability and Governance of Web and GRID Resources in Functional Genomics’

**DOI:** 10.1002/cfg.488

**Published:** 2005

**Authors:** Paul van der Vet, Theo Huibers, Pierre-Alain Binz, Martin Hofmann

**Affiliations:** 1 Department of Computer Science University of Twente PO Box 217 AE Enschede 7500 The Netherlands; 2 KPMG Business Advisory Services Amstelveen The Netherlands; 3 GeneBio Geneva Switzerland; 4 Swiss Institute of Bioinformatics Geneva Switzerland; 5 Fraunhofer Institute SCAI Sankt Augustin Germany


**Comparative and Functional Genomics**
*Comp Funct Genom* 2005; **6**: 307–310.
Published online in Wiley InterScience (www.interscience.wiley.com). **DOI:** 10.1002/cfg.488

**Conference Report**

### ESF Workshop on 'Sustainability and Governance of Web and GRID Resources in Functional Genomics'

Paul van der Vet[1]*, Theo Huibers[1,2], Pierre-Alain Binz[3,4] and Martin Hofmann[5]
[1] *Department of Computer Science, University of Twente, Enschede, The Netherlands*
[2] *KPMG Business Advisory Services, Amstelveen, The Netherlands*
[3] *GeneBio, Geneva, Switzerland*
[4] *Swiss Institute of Bioinformatics, Geneva, Switzerland*
[5] *Fraunhofer Institute SCAI, Sankt Augustin, Germany*

**Correspondence to:*
*Paul van der Vet, Department of Computer Science, University of Twente, PO Box 217, 7500 AE Enschede, The Netherlands.*
*E-mail: p.e.vandervet@utwente.nl*

Received: 17 June 2005
Revised: 23 June 2005
Accepted: 24 June 2005

### Introduction

Life scientists and bioinformaticians increasingly rely on web-based resources. The number of such resources, as well as the amount of available content, grows continuously. The need for efficient interoperability is becoming important. Moreover, as data propagate easily through such media, their quality and pertinence need to be monitored closely. In the context of the ESF Programme *Integrated Approaches to Functional Genomics*, two workshops have been organized to discuss the use of these resources, in particular for data integration (Binz *et al.*, 2002; Binz *et al.*, 2004). In both, but particularly during the latter (Geneva, October 2003), it turned out that the sustainability and governance of web-based resources have become urgent issues. It costs money to set up and maintain a resource. Because biology, like any other field of scientific enquiry, is very dynamic, maintenance is labour-intensive. Users of a resource will generally want to be assured of its quality; in other words, a scheme of quality assurance has to be in place. Governance, or who is responsible for what, has to be clear for the community. A business model addresses these issues in their mutual dependence. Therefore, every resource comes with a business model, irrespective of whether or not its creators are aware of it.

To address the issues involved, a follow-up workshop was held at the Fraunhofer Institute at Schloss Birlinghoven, Sankt Augustin, Germany, in May 2005, hosted by Martin Hofmann. The participants included the organizers of the former workshops, researchers involved in offering and/or using web services, representatives of the publishing industry, a representative of a national funding body and a representative of an industrial private enterprise. We chose to deviate from the standard workshop format of having talks followed by discussions and adopted a way of working that had all participants actively involved in exploring the issues. The outcome, unanimously agreed upon by the participants, can be summarized in one sentence: the risks involved in the current situation are so large that guidelines and actions are urgently required.

Copyright © 2005 John Wiley & Sons, Ltd.

In the invitation, the issue of business models was underlined. Briefly (actually, more a caricature), a business model explains how the mission of an organization is accomplished. It states how incomes and expenditures are matched; it identifies risks and offers strategies to deal with them. Making a profit is not an essential ingredient of a business model. Every group, institute or company has a way to balance incomes and expenditures and to address risks, but normally only commercial firms make this model explicit. We advocate that web resource providers in bioinformatics also make their business models explicit, as a first step towards ameliorating the current high-risk situation.

Business models for resources are required to address at least the following issues: quality assurance, accessibility over particular time frames (which may range from a few years to, perhaps, decades), pricing, financing, risks, and control. These issues are addressed explicitly or implicitly by any organization that operates a resource — academic institutes or societies as well as businesses. Since businesses have been operating with business models for a long time, academics might learn from their experience. Ongoing discussions on using GRID technology for eSciences underline the need for new business models for distributed knowledge resources. The workshop aimed to identify possible business models to further the ideal of a European information infrastructure for the life sciences.

The main risk for most academic resource providers is lack of funding. Their resources are created in the course of funded projects, but the very idea of project funding is at odds with sustainability because a project, by definition, has a limited lifetime, while the resource is expected to last longer than that. Since no resource can be maintained without costs, continued availability requires a source of income. This is a difficult issue because the major funding bodies tend to give priority to funding the generation of experimental data, rather than to funding the structured storage of data and information in public databases, even if a portion of the budget can be allocated to the construction of such resources. Dissemination and maintenance of the information generated in the course of a functional genomics project become problems when the project stops. Quite a number of projects have already ceased or are approaching the end of their funding period. Data, sometimes valuable data, may be lost because there is no follow-up grant or other source of income to safeguard the continued availability and maintenance of the resource. In this way, EU-funded research in functional genomics faces destruction of capital on an unprecedented scale.

### The sessions

### Session 1: exploration of the field

On this opening day, we wanted to get a better grip on the subject of resources in functional genomics and their aspects (**Martin Hofmann** and **Paul van der Vet**). The variety of biological information available over the Web proved to be too large to admit of an adequate systematization within an afternoon — sequences, SNPs, structures, interactions, pathways, metadata, ontologies, images, literature and more. As pointed out by **Amos Bairoch** and others, from a costing point of view, there are roughly two types of data resources: repositories and curated databases. Their cost structures are entirely different. In particular, the costs for curated databases are huge compared with those of a repository, and consist largely of personnel costs. Of course, this human activity is what makes these resources so valuable.

### Session 2: business models

The second day was devoted to business models. There were four introductions by speakers who are all stakeholders in one way or another.

**Martin Hofmann** (SCAI, Fraunhofer Institute, Sankt Augustin) discussed resources from the creator perspective. He provided an example of a combined wet lab/*in silico* experimental set-up that generates data believed to be of interest to others. He also drew attention to the growing importance of clinical data. The more complex a biological phenomenon is, the more likely it is that one can find it in natural-language texts, because more complex phenomena need lots of context.

**Henning Hermjakob** (EBI, Cambridge, UK) outlined the way EBI finances the often excellent resources they offer. He was aware of the dangers of project funding for infrastructure and cited the example of the resource BIND, which had to change its mode of operation drastically as a result

Copyright © 2005 John Wiley & Sons, Ltd. 

of lack of money. Quite apart from the direct disinvestment, there is indirect disinvestment which tends to be overlooked: the curators had to be laid off. Training a curator takes roughly a year or more, so laying off trained curators constitutes a large source of disinvestment.

**Geoffrey Adams** (Elsevier Science) presented an overview of web resource sustainability from a business perspective, systematizing the various components that make up a resource and discussing their financial aspects. He also warned scientists not to become addicted to funding, because funding may disappear, e.g. when EU research priorities change.

Finally, **Bernd Hägele** (Swiss Federal State Secretariat for Education and Research) provided an instructive but unfortunately rare example of cooperation between a funding body and resource maintainers. The Swiss government funds the Swiss Institute for Bioinformatics (SIB), not only because of the perceived quality of services and research it offers, but also because of its scientific importance and because it contributes to the visibility of Switzerland as a scientific country. The situation can be considered stable over the medium term.

In the second part of this session, participants divided into groups. Each group was asked to design a business model for a resource of their own choosing, so long as it dealt with content relevant to functional genomics researchers. More specifically, we asked each group to identify the services and products offered, the customers of such products/services, and the stakeholders. In addition, we asked them to identify the main cost drivers, what customers might be prepared to pay for, and reasonable revenue models. Each group reported their conclusions to the full workshop at the end of this session. One of the more striking observations was that almost all groups involved in setting up and maintaining data resources are also heavy users of such resources. This shows that there is a tight network of resources, and the loss of one of the nodes may well bring extra costs for the other nodes. To the surprise of quite a few participants, the circle of stakeholders proved to be quite large.

### Session 3: drawing lessons and identifying possible actions

The last session was a round table discussion about actions that could and should be undertaken. As stated above, all participants agreed that the current situation is simply too risky to be allowed to continue. The costs of resource providers are high and include not only maintaining and improving the resources but also searching for funds. Maintenance of the current status is unlikely in the majority of cases, as witnessed by the case of BIND. A number of factors that contribute to this instability were discussed.

First, as already mentioned, most resources are established using project money, but the duration of a project is typically less than that of a resource.

Second, funding for infrastructure is not commonplace. From the funder perspective, funding resources would mean at least a partial deviation from the current practice of project funding. This has given rise to the practice of funding infrastructure in disguise, i.e. as if it were a new project. There are exceptions: the current surge of interest in, and hence funding of, GRID technology may be interpreted as funding infrastructure. This is not entirely true, however: from the funder perspective these projects are probably regarded as seed money to develop the field and once it is mature, GRID funding as such will cease.

Third, unlike practitioners in some other fields, researchers in the life sciences expect IT/web resources to be free. This is an impediment to a system in which users of resources pay. The US National Institutes of Health, however, tends to favour user payment.

Fourth, resources grow and mature. Most resources are initially built because of prospective interest. Either creation and initial maintenance of the resource is not funded at all or it is part of a funded project that uses the resource as means of communication between the project partners. When the resource proves sufficiently interesting to third parties, a growth stage begins. The technical infrastructure is consolidated and access to the resource is improved. Data are added and a quality assessment procedure is put in place. When third parties continue to be interested in the resource, it enters the third stage, maturity. This may be accompanied by the incorporation of the resource into the service portfolio of an institute or company, either an existing one or one created specifically around the resource. To repeat what we said earlier, no resource can survive without income (if income is not visible, this can only mean that it is hidden by incomplete or inaccurate accounting). Therefore,

Copyright © 2005 John Wiley & Sons, Ltd. 

each of the three steps we have identified may end with continuation or not. Ideally, quality and scope are the factors that determine survival but in reality, of course, other factors play a role that in some circumstances may be more important than quality and scope.

Considering these four major issues and the current EU policies and funding mechanisms, the model whereby users pay was judged an alternative to the current situation that merits serious consideration. Users' costs can be covered by including a sum for use of the resource in the budget of funding proposals. The EU and other funding bodies may promote this. This would also make it possible to have resources maintained by commercial publishers (Elsevier Science, John Wiley & Sons) or semi-commercial publishers (such as learned societies), who have far more experience in cost-effective web resource management than academic groups.

However, if users are prepared to pay at all, they will normally be prepared to pay for mature resources only. It thus turns out that a resource is at its most vulnerable in the growth stage. This is the stage in which funding bodies play a decisive role. They might design guidelines that take issues such as viability, scientific quality, scope of the resource, size of the intended audience and other factors into consideration. A discussion about these matters would be helped enormously if there were some kind of business model that also outlines the long-term perspectives of the resource.

For any alternative to the current situation one may envisage, it is urgent that explicit business models are drawn up by resource providers. It would be nice if a platform were created for resource providers to share experiences and help each other with business models. As we have seen, resource providers themselves are heavy users of resources. Sustainability thus certainly constitutes a shared interest. Business models can serve as concrete anchors for a discussion between stakeholders about the future of the resources.

### References

Binz P-A, Martin A, Taussig M, de Daruvar A. 2002. Conference report. The ESF Programme on Functional Genomics Workshop on 'Data Integration in Functional Genomics and Proteomics'. *Comp Funct Genom* **3**: 16–21.

Binz P-A, Hermjakob H, van der Vet P. 2004. The ESF Programme on Functional Genomics Workshop on 'Data Integration in Functional Genomics: Application to Biological Pathways'. *Comp Funct Genom* **5**: 148–155.

Copyright © 2005 John Wiley & Sons, Ltd. 
